# Cytokine-induced killer (CIK) Cells-associated transcriptome signature reveals the potential immunomodulatory role of TNFSF14 in clear cell renal cell carcinoma

**DOI:** 10.1007/s00262-026-04422-y

**Published:** 2026-05-20

**Authors:** Yinhao Chen, Linli Chen, Xinyue Qin, Yutao Li, Di Wu, Maria Fitria Setiawan, Veronika Lukacs-Kornek, Manuel Ritter, Amit Sharma, Ingo G. H. Schmidt-Wolf

**Affiliations:** 1 Department of Integrated Oncology, Center for Integrated Oncology (CIO) Bonn, University Hospital Bonn, Venusberg Campus 1, 53127 Bonn, Germany; 2 Department of Laboratory Medicine, Affiliated Hospital of Nantong University, Medical School of Nantong University, Nantong, China; 3 Department of Hepatobiliary and Pancreatic Surgery, Affiliated Hospital of Nantong University, Nantong, 226001 China; 4 Institute of Molecular Medicine and Experimental Immunology, University Hospital of the Rheinische Friedrich-Wilhelms-University, 53127 Bonn, Germany; 5 Department of Urology and Paediatric Urology, University Hospital Bonn, Bonn, Germany

**Keywords:** Clear cell renal cell carcinoma, Cytokine-induced killer cell, Risk stratification, Machine learning, Immunotherapy

## Abstract

Among adoptive immune cell therapies, cytokine-induced killer cell (CIK) therapy has demonstrated clear therapeutic relevance in multiple cancers, particularly clear cell renal cell carcinoma (ccRCC). Despite being clinically successful, the molecular signatures associated with the immune-regulatory role of these CIK cells in cancers remain elusive. Considering this, we systematically identified CIK expansion-induced genes (CIK-EIGs) from their transcriptome profiles and curated the CIK-EIG-anchored transcriptome framework across bulk and single-cell ccRCC datasets. Using comprehensive bioinformatics analysis, we next developed the CIK-EIG-anchored risk stratification (CIKRRS), which enabled reliable prediction of patient prognosis, correlated with the clinical-pathological course, and defined an immunologically inflamed but clinically unfavorable phenotype. Of interest, the integrative analyses highlighted TNFSF14 as one of the key functional modulators within the CIK-EIG-associated immune landscape, showing a strong association with cytotoxic immune signaling and cancer-immunity cycle activity. Subsequently, the functional assays verified that TNFSF14 significantly enhanced CIK-mediated tumor cell killing, degranulation, and cytokine production in ccRCC cell lines, confirming its role in enhancing CIK effector function. Taken together, our findings present a CIK-EIG-anchored transcriptomic and prognostic framework and highlight the potential relevance of TNFSF14 for further optimization of CIK-based immunotherapy in ccRCC patients.

**Supplementary Information:**

The online version contains supplementary material available at 10.1007/s00262-026-04422-y.

## Introduction

Clear cell renal cell carcinoma (ccRCC) stands out from other kidney cancers due to its aggressive nature and tendency to metastasize, leading to a poor prognosis and high mortality [1]. ccRCC is notably resistant to conventional treatment modalities, including radiotherapy and chemotherapy [2]. As our knowledge of the molecular pathology of ccRCC expands, new targeted therapies and immunotherapies are being developed, showing considerable promise in improving outcomes for patients with advanced disease [3]. Moreover, the tumor microenvironment significantly influences ccRCC development and its response to therapy. Integrating relevant stromal and immune biomarkers may yield effective predictive and prognostic features, thereby informing the management of current therapeutic strategies and guiding future drug development [4].

Cytokine-induced killer (CIK) cells were first characterized by Schmidt-Wolf et al. [5] They were generated through in vitro expansion and represent a heterogeneous immune cell population mainly composed of CD3⁺CD56⁺ NK-like T cells, along with various T cell subsets and a small proportion of NK cells [6, 7]. These cells exhibit MHC-unrestricted antitumor activity [8, 9], a feature attributed to the activation of natural killer group 2 member D receptors, which mediate cytotoxicity against a broad spectrum of hematologic and solid malignancies, including renal carcinoma [10]. Moreover, CIK cells are known to produce critical cytokines, including IFN-**γ** and interleukin-6, which amplify their antitumor efficacy [11]. Notably, CIK cell-based immunotherapy has demonstrated a substantial extension of overall survival compared to conventional therapies [12, 13]. Recently, CIK cell therapy has been investigated for more than three decades and has obtained regulatory approval in several countries, including Germany [14, 15]. In addition, CIK cells and their derivatives, including dendritic cell-CIK combinations (CIK-DCs), have been proposed as an effective adjuvant therapy in the treatment of RCCs [10, 16, 17]. In this study, we used transcriptomic changes induced during ex vivo CIK generation as a reference framework. Specifically, we defined a gene set termed CIK expansion-induced genes (CIK-EIGs) from Day 14 mature CIK cells versus Day0 PBMC during CIK expansion.

**Fig. 1:**
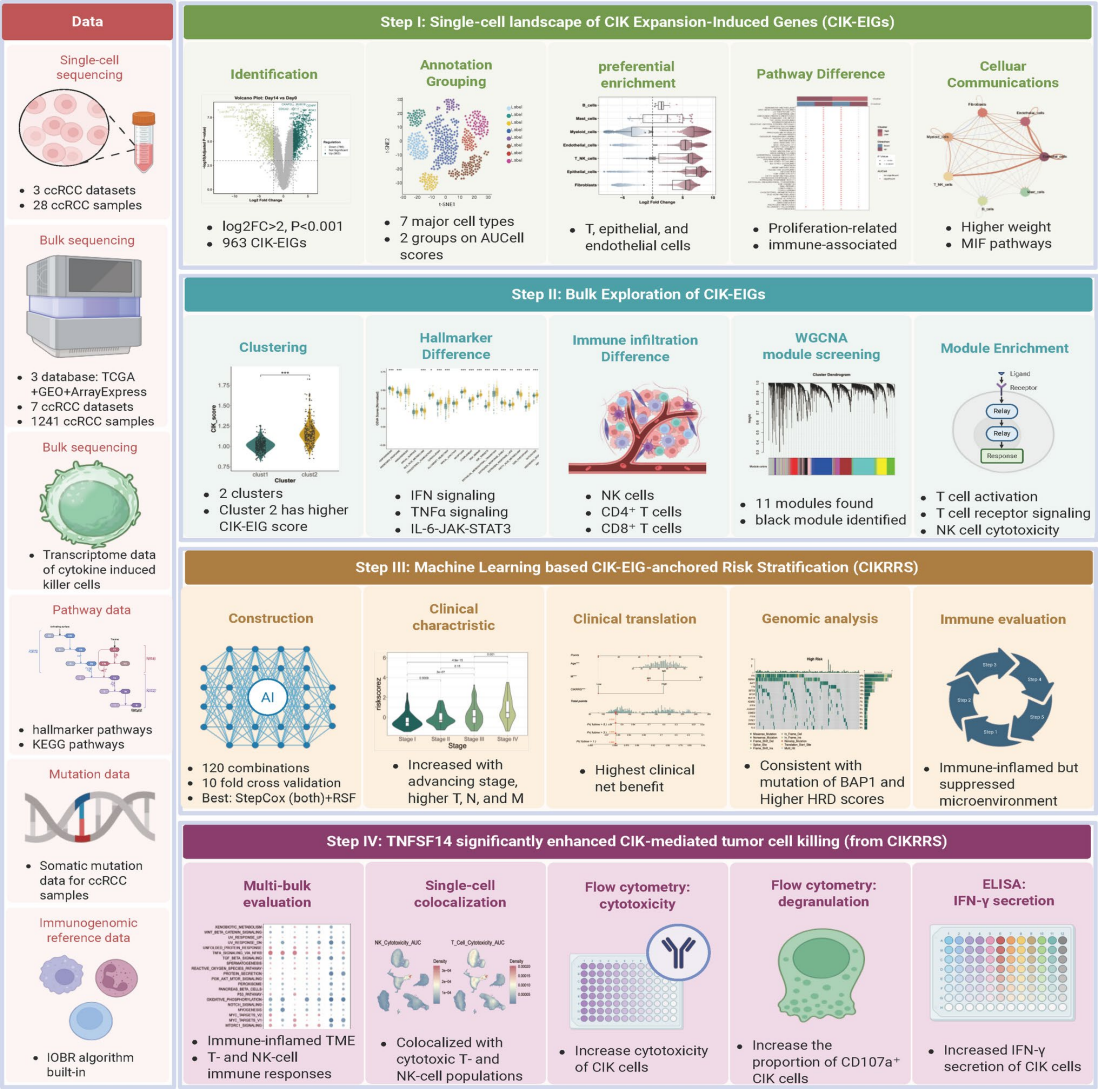
Whole workflow of this study

Building on this framework, we conducted a comprehensive multi-level transcriptomic analysis leveraging publicly available bulk and single-cell ccRCC datasets to delineate CIK-EIG-anchored immune-inflamed heterogeneity across patients. Using weighted gene co-expression network analysis (WGCNA), we identified prognostically relevant gene modules and developed a CIK-EIG-anchored risk stratification system (CIKRRS). To enhance robustness and clinical relevance, CIKRRS was constructed using multiple survival-oriented machine learning algorithms and model combinations and evaluated across prognostic performance, mutational characteristics, immune landscape, and drug sensitivity. Finally, we performed functional assays to examine the impact of TNFSF14, a key component gene of CIKRRS, on CIK cell cytotoxic activity. The study design and analytical workflow are outlined in Fig. 1.

## Methods

### Data collection and organization

The bulk RNA-sequencing datasets of ccRCC included TCGA-KIRC (*n* = 525), E-MTAB-1980 (*n* = 101), E-MTAB-3218 (*n* = 114), GSE126964 (*n* = 55), GSE40435 (*n* = 101), GSE53757 (*n* = 72), and GSE73731 (*n* = 265). Among these, TCGA-KIRC and E-MTAB-1980, which contain relatively comprehensive clinical survival information, were used for model construction and validation. The expression profiling data of CIK cells at different time points were obtained from Meng et al. [18]. Furthermore, we downloaded TCGA’s somatic mutation data in the Mutation Annotation Format (MAF). HRD-related scoring data and cytolytic activity (CYT) data were downloaded from the previous studies [19, 20]. Single-cell RNA-sequencing data for ccRCC were sourced from the GEO database, including GSE156632, GSE171306, and GSE242299, which comprised 7, 2, and 19 ccRCC samples, respectively.

### Single-cell analysis

Following quality control, with criteria of 200—6,000 genes per cell and mitochondrial gene expression under 20% per sample. Using DecontX to remove ambient RNA contamination, the processes of normalization, dimensionality reduction, clustering, and cell-type annotation were executed using the Seurat package [21]. Cell-type enrichment was assessed using observed-to-expected (Ro/e) ratios [22] derived from single-cell contingency tables and visualized with ComplexHeatmap, while differential neighborhood abundance was analyzed using miloR [23]. AUCell package was applied to evaluate the activity of hallmarker gene sets or a custom gene set. We also explored cellular interactions using the R package CellChat comparing ligand–receptor interactions to assess the impact of CIK-EIGs at the single-cell level [24].

### Identification of CIK-EIGs and screening of model-building genes

Differential expression analysis was performed on an ex vivo CIK generation transcriptome dataset from Meng et al. (Day0 PBMC vs Day14 mature CIK cells; *n* = 4 per group) [18]. Expression values (RPKM) were log2-transformed [log2(RPKM + 1)] and analyzed using the limma package with empirical Bayes moderation. P values were adjusted for multiple testing using the Benjamini–Hochberg false discovery rate (BH-FDR). Genes significantly upregulated in Day14 versus Day0 (log2FC > 2 and BH-FDR < 0.001) were defined as CIK-EIGs. CIK-EIGs capture a transcriptional program induced during ex vivo CIK generation and are used as a reference to map similar activation programs in tumor datasets; however, they do not constitute direct evidence of bona fide CIK cells residing in untreated ccRCC tumors. Subsequently, consensus clustering was executed with the help of the ConsensusClusterPlus package in R [25]. Moreover, WGCNA and correlation analysis were applied to retrieve modules associated with poorer prognostic clustering [26]. The intersection of differentially expressed genes in tumor tissues in TCGA-KIRC and key module genes was performed. The result of Cox regression analyses for these genes was utilized in subsequent model construction. In tumor bulk and single-cell datasets, CIK-EIG activity score was quantified using signature scoring and downstream association analyses rather than re-deriving CIK-EIGs through differential expression testing.

### Construction of machine learning-based risk stratification

The TCGA-KIRC cohort was assigned as the training set, while the E-MTAB-1980 cohort served as an independent external validation set. To ensure robust prognostic model development, we applied multiple survival-oriented machine learning algorithms (Table S1). Model development and selection were performed exclusively within the training cohort using tenfold cross-validation, with Harrell’s concordance index (C-index) as the primary metric. The selected modeling framework was then locked and evaluated in the external validation cohort without any re-fitting. Risk scores derived from the locked model were subsequently used for survival analyses and downstream investigations.

### Survival analysis and relationship to clinical characteristics

Subsequently, patients were classified into high- and low-risk groups based on their CIKRRS risk scores, followed by Kaplan–Meier (KM) analysis for overall survival (OS). For KM visualization, patients were dichotomized using the cohort-specific median CIKRRS within each dataset to provide an interpretable, distribution-anchored stratification. In parallel, CIKRRS was evaluated as a continuous variable in Cox regression models and by time-dependent ROC analysis using the timeROC package [27]. We further examined the association between CIKRRS and clinicopathological characteristics in the TCGA-KIRC cohort, assessed its stability through subgroup survival analyses, and compared its prognostic accuracy with conventional clinical variables. Univariate and multivariate Cox regression analyses were performed to determine whether CIKRRS is an independent prognostic factor, and a nomogram was constructed accordingly. The nomogram was evaluated using calibration curves and decision curve analysis (DCA). To further evaluate whether CIKRRS merely reflects generic cytotoxic activity, we performed an additional multivariable Cox regression analysis including both CIKRRS riskScore and CYT in the TCGA-KIRC cohort.

### Enrichment analysis

Using ssGSEA in the GSVA package, we calculated CIK-EIG and pathway activity scores in TCGA-KIRC samples [28]. Fifty Hallmark pathways (h.all.v2024.1.Hs.symbols.gmt) were analyzed with Gaussian kernel estimation. KEGG, GO, and GSEA were performed using clusterProfiler, with adjusted *P* < 0.05 considered significant.

### Immunological characteristics assessment

Immune, stromal, and tumor purity scores were calculated using estimateScore [29]. Tumor microenvironment cell proportions were estimated with deconvo_tme in IOBR using CIBERSORT [30], EPIC [31], MCPcounter [32], and TIMER [33]. The combined dataset was standardized for downstream analyses. Cancer-immunity cycle activity in TCGA-KIRC was evaluated using the Tracking Tumor Immunophenotyping (TIP) platform.

### Genomics analysis and treatment predictions

Somatic mutation landscapes of high- and low-risk ccRCC patients were visualized using maftools [34]. TMB was defined as the number of mutations per megabase in exonic regions [35]. HRD status was assessed using HRD_TAI, HRD_LST, and HRD_LOH, and the overall HRD score was calculated as their sum [19]. pRRophetic was used to estimate drug sensitivity in patients with different CIKRRS scores by predicting IC50 values from gene expression profiles [36].

### TNFSF14 stratification and downstream analyses

For TNFSF14-stratified GSEA, samples in each bulk dataset were ranked by TNFSF14 expression, and the top and bottom 30% were defined as TNFSF14-high and TNFSF14-low groups. Differential expression between groups was analyzed with limma to generate a pre-ranked gene list (log2FC), followed by GSEA with BH adjustment. For correlation-based analyses, TNFSF14 expression was treated as a continuous variable, and associations were assessed by Spearman’s correlation. For prognostic benchmarking, TNFSF14 was also modeled as a continuous variable.

## Cell culture

CIK cells were generated and cultured as previously described [5]. PBMCs were provided by the blood bank of the University Hospital Bonn and isolated by density gradient centrifugation using Pancoll (Pan-Biotech, Aidenbach, Bavaria, Germany). Human renal cancer cell lines 769-P and 786-O were acquired from the American Type Culture Collection located in Manassas, Virginia, USA. Before conducting experiments, all cell lines were regularly checked and verified to be free of mycoplasma using a detection kit from Thermo Fisher Scientific in Darmstadt, Germany. In RPMI 1640 medium (PAN-Biotech, Aidenbach, Germany) containing 10% FBS (Gibco, Munich, Germany), 100 U/mL penicillin, and streptomycin (Gibco, Munich, Germany), tumor cells were kept at 37° C with 5% CO_2_.

### Antibodies and reagents

Recombinant human TNFSF14 (LIGHT) protein, anti-human TNFSF14 neutralizing antibody (clone 115,520, mouse IgG1), and the corresponding mouse IgG1 isotype control (clone MOPC-21) were purchased from R&D Systems (Minneapolis, MN, USA). BioLegend (San Diego, CA, USA) provided the fluorochrome-conjugated monoclonal antibodies for flow cytometry along with the corresponding isotype controls, including mouse anti-human CD3-FITC (clone OKT3), CD4-APC (clone OKT4), CD56-PE (clone 5.1H11), CD8a-Brilliant Violet 421 (BV421; clone RPA-T8), CD56-FITC (clone HCD56), CD3-APC (clone UCHT1), and CD107a-PE (clone H4A3). 7-Aminoactinomycin D (7-AAD) was used as a viability dye (PerCP-7-AAD). Cytokines such as IFN-γ, IL-2, and IL-1β were sourced from ImmunoTools located in Aidenbach, Bavaria, Germany.

### Flow cytometric phenotyping of CIK cells

Following a 14-day in vitro expansion period, CIK cells were collected, rinsed twice with phosphate-buffered saline (PBS), and then resuspended in 100 μL of FACS buffer at a concentration of 1 × 10^7^ cells/mL. Cells underwent a 20-min incubation on ice in the dark with fluorochrome-conjugated monoclonal antibodies (1 μg/mL) against CD3-FITC, CD8a-BV421, CD56-PE, and CD4-APC. Corresponding isotype controls were included to define gating thresholds. Once stained, the cells underwent two PBS washes and were incubated with 7-AAD at room temperature for 10 min in the dark to remove non-viable cells. Immediately, the samples underwent testing on a BD FACSCanto II flow cytometer manufactured by BD Biosciences in Heidelberg, Germany.

### Cytotoxicity assay

CIK cytotoxicity against renal cancer cells was assessed by flow cytometry as previously described [37]. Target cells labeled with CFSE (5 μM, 37° C, 15 min) were placed in 96-well plates (4 × 10^4^ cells per well) and co-cultured with CIK cells at a 10:1 effector-to-target ratio under specified conditions, including TNFSF14 stimulation (100 ng/mL) and antibody blockade or isotype control (1 μg/mL). Following a 24-h incubation at 37° C, cells were gently removed with Accutase (BioLegend, Koblenz, Germany), washed, and stained with Hoechst 33,258 (1 μg/mL; Cayman Chemical, Hamburg, Germany) to mark dead target cells. Samples were acquired on a BD FACSCanto II flow cytometer and analyzed using FlowJo v10. Cytotoxicity (%) was calculated as ((CL − TL)/CL) × 100%, where CL and TL denote viable CFSE⁺ target cells in control and treated wells, respectively.

### CD107a degranulation assay

CIK cell degranulation was assessed by surface CD107a expression as previously described [38]. In the presence of anti-CD107a-PE (1 μg/mL; BioLegend) and brefeldin A (10 μg/mL), CIK cells were co-cultured with renal cancer cells at a 10:1 effector-to-target ratio for 4 h. Using 7-AAD, dead cells were excluded, and the remaining cells were stained with anti-CD3-APC and anti-CD56-FITC before being acquired on a BD FACSCanto II flow cytometer.

### ELISA for IFN-γ

CIK cells and renal cancer cells were co-cultured at a 10:1 effector-to-target ratio for 24 h under the specified treatment conditions. According to the manufacturer’s instructions, cell-free supernatants were obtained, and IFN-γ concentrations were determined using a commercial ELISA kit (Invitrogen, Camarillo, CA, USA).

### Statistical analysis

Statistical analysis, visualization, and data processing were performed with R4.3.1. The Wilcoxon rank sum test was employed for comparing two groups in bioinformatics analysis. Correlations were analyzed using Spearman’s rank coefficient, while survival curves were assessed with the log-rank test. FlowJo v10.6 (FlowJo, LLC, Ashland, OR, USA) facilitated the analysis of flow cytometry data, while GraphPad Prism v8.0 (GraphPad Software, San Diego, CA, USA) was utilized for conducting statistical analyses. All CIK cell-related experiments were performed with cells derived from at least three independent biological donors. Mean ± SD is used to present the quantitative data. One-way or two-way ANOVA was employed for group comparisons, and Bonferroni’s post hoc test was conducted subsequently. A p value under 0.05 indicated statistical significance. For high-dimensional analyses such as differential expression and pathway enrichment, multiple testing was controlled using the Benjamini–Hochberg FDR procedure unless otherwise stated.

## Results

### CIK-EIGs define an ex vivo CIK expansion-induced proliferative and cytotoxic program at single-cell resolution

**Fig. 2:**
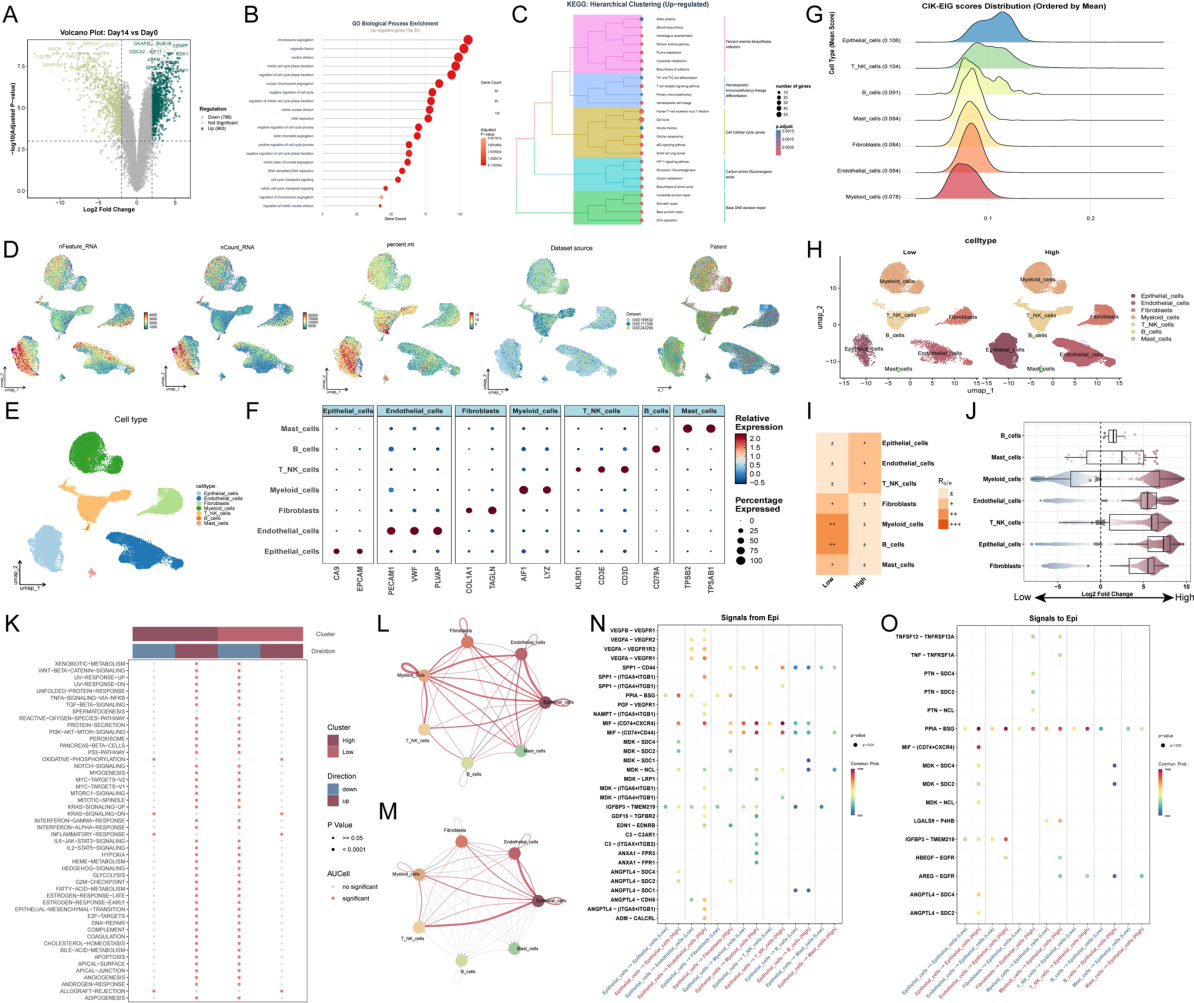
CIK-EIGs-associated landscape on single-cell level **A** Volcano plot showing differentially expressed genes between Day 14 and Day 0 CIK cells (log2FC > 2, adjusted *P* < 0.001), defining CIK expansion-induced genes (CIK-EIGs). **B** GO Biological Process enrichment analysis of CIK-EIGs based on adjusted *P* values. **C** KEGG pathway enrichment analysis of CIK-EIGs based on adjusted *P* values. **D** UMAP visualization of integrated single-cell RNA-seq datasets colored by quality control metrics, dataset source, and patient, showing no evident batch effects. **E** UMAP annotation of seven major cell types based on classical markers. **F** Dot plot of representative marker genes used for cell-type identification; color indicates scaled average expression, and dot size indicates the percentage of expressing cells. **G** Distribution of AUCell-derived CIK-EIG scores across cell types at the single-cell level. **H** UMAP projections of single cells from samples stratified into CIK-EIG score-high and CIK-EIG score-low group. **I** Group preference of individual cell clusters estimated by the Ro/e score. + + + , Ro/e > 2.0; + + , 1.5 < Ro/e ≤ 2.0; + , 1.0 ≤ Ro/e ≤ 1.5; ± , 0 < Ro/e < 1.0; − , Ro/e = 0. **J** Beeswarm and box plots showing the distribution of log2-fold differences in neighborhoods in different cell-type clusters. **K** Hallmark pathway activity analysis based on AUCell scores comparing CIK-EIG scores high and low samples; dot size indicates significance and color indicates direction of change. **L**, **M** Cell–cell communication inferred by CellChat comparing CIK-EIG score groups from count-based **L** and weight-based **M** perspectives. The color of the edge represents a change in trend; red indicates an increase in communication in the high CIK-EIG score group, while blue indicates the opposite. **N** Differential outgoing ligand–receptor signaling from epithelial cells to other cell types. **O** Differential incoming signaling to epithelial cells

Through differential expression analysis of ex vivo CIK generation (Day 14 vs Day 0), a total of 963 CIK-EIGs were identified (Fig. 2A**, **Table S2). Further enrichment analyses indicated that these genes were primarily associated with pathways linked to cell growth and cytotoxic activities, such as the regulation of cell cycle phase transitions, T cell receptor signaling, DNA replication, Th1 and Th2 cell differentiation, and primary immunodeficiency (Fig. 2B-C).

To characterize the single-cell landscape of CIK-EIGs, single-cell RNA-seq data from three independent datasets were integrated and analyzed. UMAP visualization revealed well-defined cellular populations, with comparable distributions of quality control metrics across clusters and no evident batch effects among datasets or patients (Fig. 2D). Then, cells were classified into seven major cell types, including epithelial cells, endothelial cells, T cells, B cells, fibroblasts, myeloid cells, and mast cells (Fig. 2E), which was further validated by classical marker gene expression patterns (Fig. 2F). CIK-EIG scores were subsequently calculated at the single-cell level using AUCell based on CIK-EIGs. The distribution of CIK-EIG scores varied across cell types, with relatively higher scores observed in epithelial cells and T cells (Fig. 2G). Notably, the CIK-EIG score reflects activation of an ex vivo expansion-induced program and does not imply CIK cell identity; elevated scores in non-immune compartments may arise from shared proliferative and IFN-related transcriptional components. We further calculated cell-level CIK-EIG AUCell scores and summarized them per tumor sample as the mean AUCell score. Samples were then dichotomized by the cohort-specific median into CIK-EIG-high and CIK-EIG-low groups (*n* = 28 tumors; 14 high and 14 low). UMAP projection revealed distinct differences in cell-type distribution between the two groups (Fig. 2H), with Ro/e and miloR analyses consistently indicating preferential enrichment of T cells, epithelial cells, and endothelial cells in the high CIK-EIG score group (Fig. 2I-J).

Hallmark pathway analysis demonstrated that CIK-EIG-high samples exhibited coordinated activation of proliferation-related programs, including E2F targets, MYC targets, G2M checkpoint, and mTORC1 signaling, together with immune-associated pathways linked to cytotoxic effector function, such as IFN responses, IL-2-STAT5 signaling, and TNFα-NF-κB signaling (Fig. 2K). These changes were collectively consistent with the proliferative and cytotoxic features observed above. Cell–cell communication analysis further revealed that epithelial cells in the high CIK-EIG score group displayed markedly stronger interactions with other cell types from both count- and weight-based perspectives, particularly with T cells and endothelial cells (Fig. 2L-M). Ligand–receptor analysis showed that epithelial cells primarily enhanced outgoing signaling toward T cells through MIF-related pathways (CD74/CXCR4 and CD74/CD44) and SPP1/CD44 interactions (Fig. 2N), whereas increased reception of T cell-derived signals was mainly mediated via the PPIA/BSG axis (Fig. 2O).

### Bulk transcriptomic profiling identifies CIK-EIG-anchored immune-inflamed subtypes

**Fig. 3:**
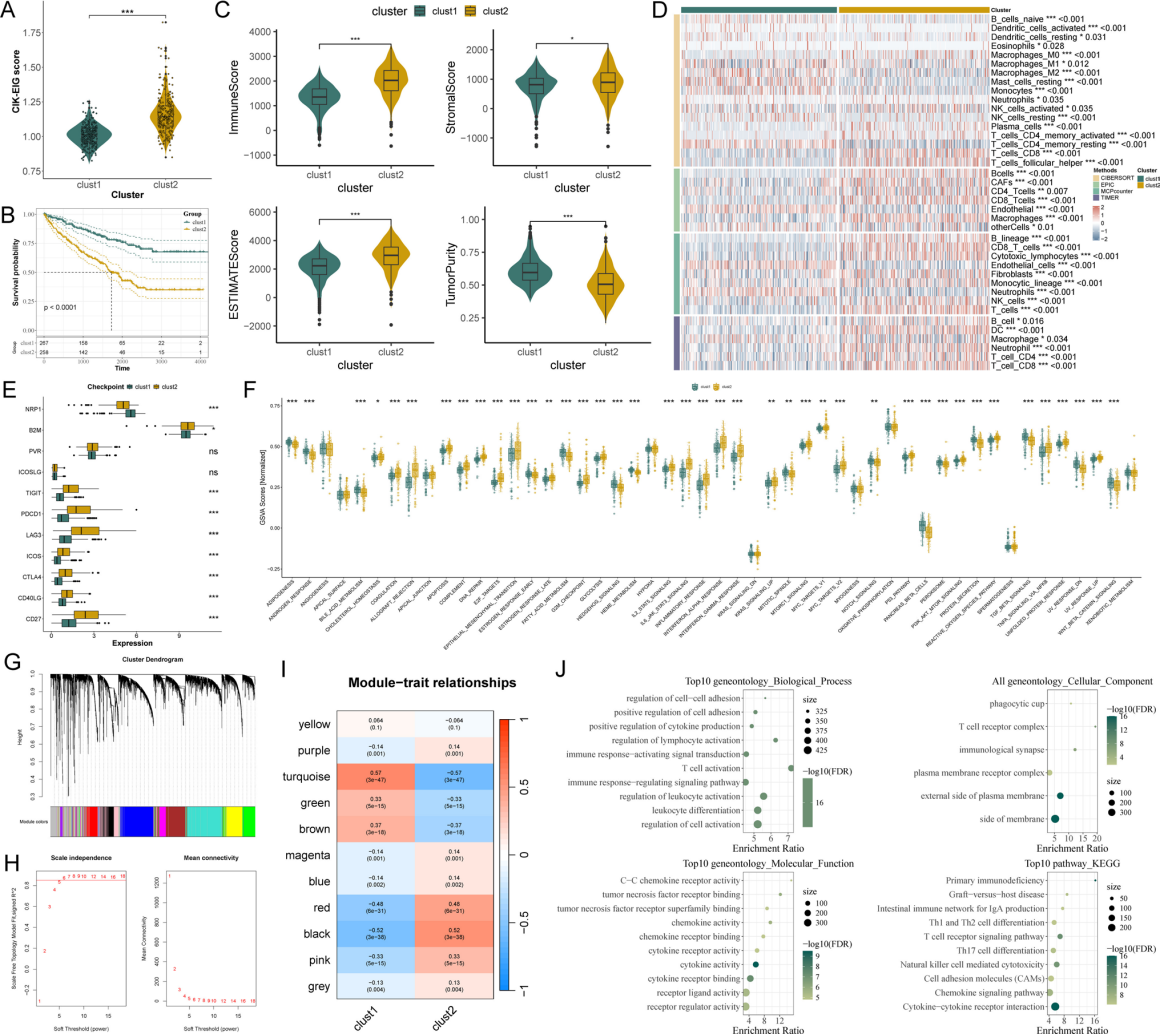
CIK-EIG-based assessment on bulk level **A** Violin plot comparing GSVA-derived CIK-EIG scores between Cluster 1 and Cluster 2 (Wilcoxon test). **B** Kaplan–Meier overall survival analysis demonstrates poorer prognosis in Cluster 2 compared with Cluster 1 (log-rank test). **C** Violin plots comparing ImmuneScore, StromalScore, ESTIMATEScore, and TumorPurity between clusters (Wilcoxon test). **D** Heatmap summarizing immune cell infiltration levels estimated by CIBERSORT, EPIC, MCPcounter, and TIMER algorithms (Wilcoxon test). **E** Box plots illustrating the expression of immune checkpoint molecules (Wilcoxon test). **F** Box plots of GSVA pathway enrichment scores, revealing enrichment of immune- and inflammation-related pathways in Cluster 2 (Wilcoxon test). **G** Scale-free topology fit index and means connectivity plots across different soft thresholding powers used for weighted gene co-expression network analysis (WGCNA), with power = 6 selected to achieve optimal scale-free topology and network connectivity. **H** Hierarchical clustering dendrogram based on topological overlap, identifying 11 gene modules represented by distinct colors. **I** Heatmap of module–trait relationships showing correlations between gene modules and clusters, with the black module displaying the strongest positive correlation with Cluster 2. **J** Functional enrichment analyses of genes in the black module, including GO biological processes, cellular components, molecular functions, and KEGG pathways, highlighting immune-related functions such as T cell activation, T cell receptor signaling, NK cell-mediated cytotoxicity, and cytokine–cytokine receptor interactions *P < 0.05, **P < 0.01, ***P < 0.001

Two stable molecular subtypes were identified in the TCGA-KIRC cohort through unsupervised clustering using CIK-EIGs (**Table S3**). Cluster 2 had notably higher CIK-EIG scores than Cluster 1 (*P* < 0.001, Fig. 3A) and was linked to worse overall survival (*P* < 0.0001, Fig. 3B). Cluster 2 showed significantly elevated immune, stromal, and ESTIMATE scores, indicating increased infiltration of immune and stromal components (*P* < 0.05, Fig. 3C). Consistently, higher levels of NK cells as well as CD4⁺ and CD8⁺ T cells were observed in Cluster 2 (*P* < 0.0001, Fig. 3D), as validated by multiple immune deconvolution algorithms, including CIBERSORT, EPIC, MCPcounter, and TIMER. In addition, expression of immune checkpoint molecules, such as CTLA4, PDCD1, and LAG3, was markedly increased in Cluster 2, suggesting an immunosuppressive tumor microenvironment (*P* < 0.001, Fig. 3E). According to GSVA, Cluster 2 showed a significant enrichment in pathways associated with immune response and inflammation, such as TNFα signaling via NF-κB, IL-6-JAK-STAT3, IL-2-STAT5, IFN signaling, and epithelial-mesenchymal transition (*P* < 0.001, Fig. 3F). In TCGA-KIRC, the bulk CIK-EIG score was positively correlated with cytolytic activity (Spearman *r* = 0.468, *P* < 0.001; Fig. S1), consistent with an immune-inflamed activation state.

To further explore the gene regulatory architecture associated with Cluster 2, WGCNA was performed (Fig. 3G). Following the removal of genes with high expression variability, a soft thresholding power of 6 was chosen to build a scale-free network, leading to the discovery of 11 gene modules (Fig. 3H). With a correlation coefficient of 0.52 and a *P* value less than 0.001, the black module exhibited the strongest positive link to Cluster 2 and was picked for further examination (Fig. 3I). The functional enrichment analysis indicated that genes within the black module were mainly associated with immune-related processes. GO analysis showed significant enrichment in immune-related terms, including T cell activation and leukocyte regulation (BP), the T cell receptor complex and immunological synapse (CC), and cytokine activity (MF), while KEGG analysis highlighted immune pathways such as natural killer cell-mediated cytotoxicity, T cell receptor signaling, and cytokine–cytokine receptor interaction (Fig. 3J).

### Development and validation of a CIK-EIG-anchored prognostic stratification

**Fig. 4:**
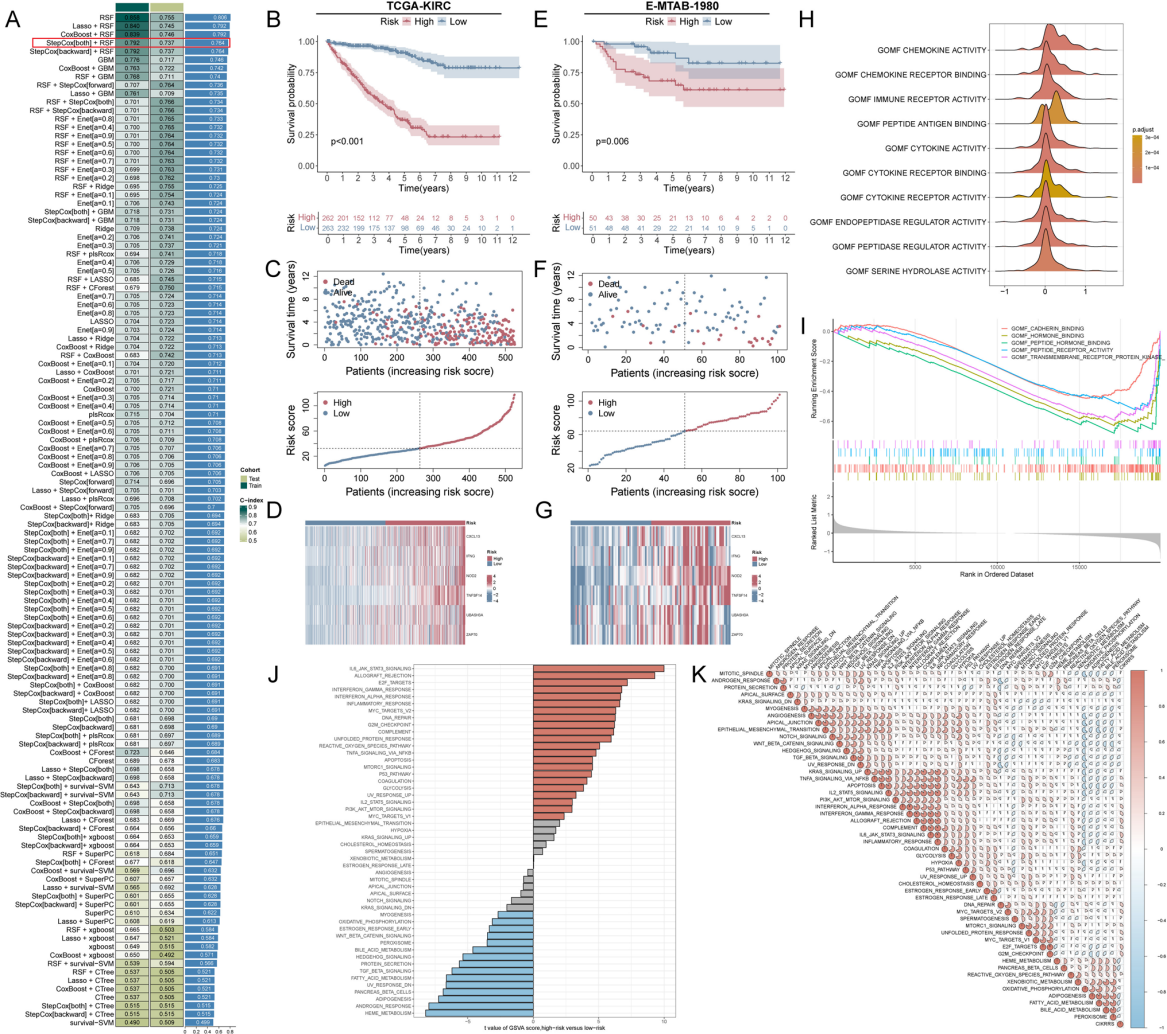
Development and validation of the CIKRRS model **A** Heatmap of C-index values for 120 machine learning model combinations constructed from black-module genes and evaluated across cohorts. The StepCox (both directions) and Random Survival Forest (RSF) model exhibited the most stable and optimal performance. **B**-**D** TCGA-KIRC cohort: **B** Kaplan–Meier overall survival curves for CIKRRS-defined high- and low-risk groups (log-rank test); **C** distribution of risk scores and corresponding survival status; and **D** heatmap of CIKRRS gene expression in high- and low-risk groups. **E**–**G** E-MTAB-1980 cohort: like **B**-**D** **H** GSEA molecular function enrichment analysis in the high-risk group, highlighting immune-related functions. **I** Molecular function enrichment analysis in the low-risk group, showing enrichment in peptide hormone binding and receptor activity. **J** GSVA Hallmark pathway scores comparing high- and low-risk groups. **K** Correlation between CIKRRS scores and GSVA Hallmark pathways; dot size and color represent correlation strength. *P < 0.05, **P < 0.01, ***P < 0.001

With 120 combinations of machine learning methods, a CIKRRS was constructed based on the univariate results of intersecting module genes and differently expressed genes in TCGA-KIRC (**Table S4**). The model integrating StepCox (both) and Random Survival Forest (RSF) was selected as a robust and high-performing model, achieving a C-index of 0.792 in the training cohort and 0.737 in the independent external validation cohort (Fig. 4A). The performance of the CIKRRS signature was validated by KM curves, risk score distributions, and survival status scatter plots (*P* < 0.01, Fig. 4B, C, E, and F). A steady pattern emerged indicating that ccRCC patients with elevated risk scores had a less favorable prognosis in both cohorts (**Table S5 and Table S6**). In the high-risk group, all CIKRRS genes showed increased expression (Fig. 4D and G).

### High-risk CIKRRS status is characterized by activated immune signaling

GSEA analyses for MF showed that high-risk ccRCC patients were significantly related to immune-associated activity, including immune receptor activity, cytokine activity, and cytokine receptor activity (Fig. 4H). Peptide hormone binding and peptide receptor activity were enriched in the low-risk group (Fig. 4I). Further GSVA assessment for Hallmark pathways demonstrated that several severe immune pathways were upregulated in ccRCC patients with high risk, including IL6-JAK-STAT3 signaling, IFN-α, and IFN-γ pathways (Fig. 4J). Subsequent correlation analysis confirmed this finding, showing significantly higher correlations between CIKRRS and the above pathways (Fig. 4K).

### Clinical relevance and prognostic value of the CIKRRS stratification

**Fig. 5:**
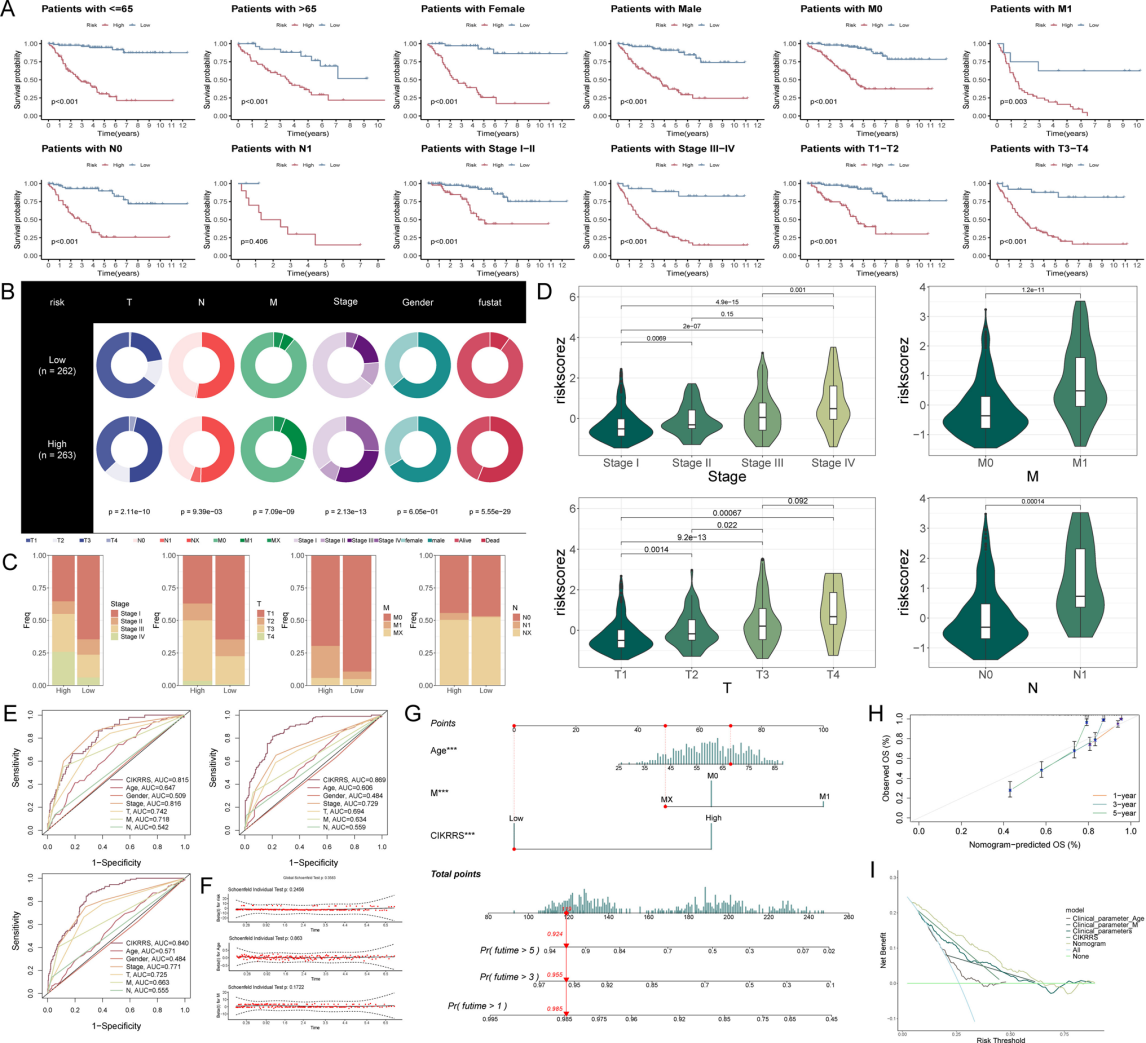
Clinical relevance and prognostic performance of the CIKRRS **A** Kaplan–Meier overall survival analyses stratified by CIKRRS risk groups across major clinical subgroups, including age, sex, and tumor stage, showing significantly poorer survival in the high-risk group except for the N1 subgroup (log-rank test). **B** Associations between CIKRRS risk groups and clinicopathological characteristics, including T stage, N stage, M stage, overall stage, and survival status, assessed by chi-square tests. **C** Stacked bar plots showing the distribution of overall stage and T/N/M classifications between high- and low-risk groups, with enrichment of advanced-stage disease in the high-risk group. **D** Violin plots of CIKRRS risk scores across overall stage (I-IV) and TNM categories, demonstrating a progressive increase in risk scores with advancing disease. **E** Time-dependent ROC curves for 1-, 3-, and 5-year overall survival comparing the predictive performance of CIKRRS with conventional clinical parameters. **F** Schoenfeld residual tests evaluating the proportional hazards assumption for age, M stage, and CIKRRS, showing no significant violations. **G** Nomogram integrating age, M stage, and CIKRRS to estimate individualized probabilities of 1-, 3-, and 5-year overall survival based on assigned scores for each predictor. **H** Calibration curves assessing nomogram performance at 1, 3, and 5 years, demonstrating good agreement between predicted and observed outcomes. **I** Decision curve analysis evaluating the clinical net benefit of the nomogram, showing superior performance compared with clinical parameters alone across a range of threshold probabilities. *P < 0.05, **P < 0.01, ***P < 0.001

Figure 5A illustrates that patients classified as high risk generally had worse overall survival in most clinical categories (*P* < 0.05), with the exception of the N1 subgroup. Clinicopathological characteristics such as N stage, M stage, T stage, overall stage, and survival status showed significant differences between the two risk groups, as demonstrated by the chi-square analysis (*P* < 0.05, Fig. 5B). Notably, patients with advanced-stage disease were disproportionately enriched in the high-risk group (Fig. 5C). CIKRRS increased progressively with advancing overall stage, as well as with higher T, N, and M categories (Fig. 5D). In predicting 1-, 3-, and 5-year overall survival, time-dependent ROC analyses revealed that the CIKRRS model consistently surpassed conventional clinical parameters in accuracy (Fig. 5E). Age, M stage, and CIKRRS were found to be independent predictors of overall survival through univariate and multivariate Cox regression analyses (Fig. S2**A-B, Table S7**), with no violation of the proportional hazards assumption as assessed by Schoenfeld residuals (Fig. 5F). A nomogram was developed to predict overall survival at 1, 3, and 5 years based on these factors (Fig. 5G), which demonstrated good predictive performance, as supported by calibration and decision curve analyses (Fig. 5H-I). Importantly, in a multivariable Cox model including both CIKRRS riskScore and CYT, riskScore remained strongly and independently associated with overall survival (HR = 1.046, 95% CI 1.040–1.053, *P* < 0.001; **Table S8**), indicating that the prognostic effect of CIKRRS is not merely attributable to generic cytotoxic activity.

### CIKRRS stratification defines therapeutically distinct tumor states

**Fig. 6:**
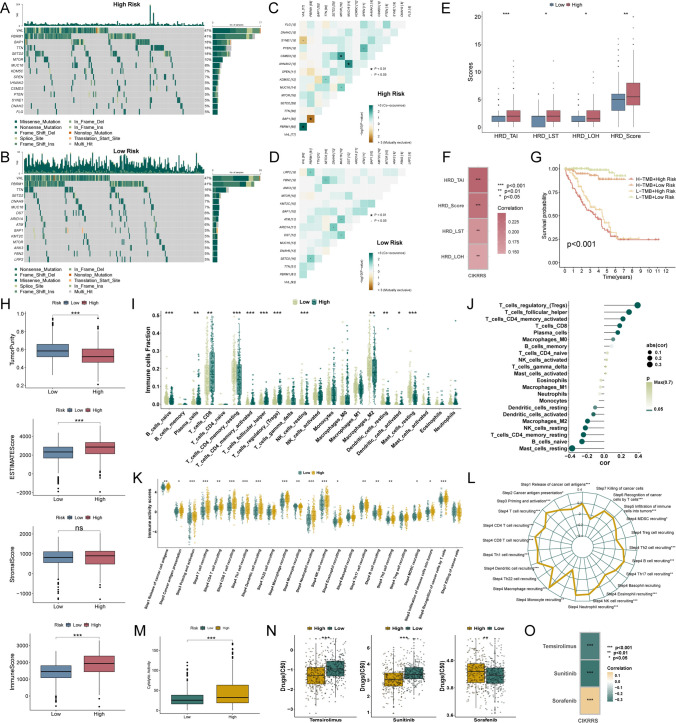
Genomic instability, immune landscape, and therapeutic implications associated with CIKRRS **A**, **B** Somatic SNV mutation landscapes of the high-risk **A** and low-risk **B** groups, showing higher mutation frequencies of BAP1, SETD2, and MTOR in high-risk patients. **C**, **D** Co-occurrence analyses of somatic mutations in high-risk **C** and low-risk **D** groups, identifying significant co-mutated gene pairs. **E** Comparison of homologous recombination deficiency (HRD) metrics, including HRD_TAI, HRD_LST, HRD_LOH, and HRD score, between risk groups (Wilcoxon test). **F** Correlation between CIKRRS scores and HRD metrics (Spearman test). **G** Kaplan–Meier overall survival stratified by tumor mutational burden (TMB) and CIKRRS risk groups, showing the poorest prognosis in patients with both high TMB and high CIKRRS (log-rank test). **H** Comparison of StromalScore, ImmuneScore, ESTIMATEScore, and TumorPurity between groups (Wilcoxon test). **I** Immune cell infiltration estimated by CIBERSORT, showing increased proportions of plasma cells, CD8⁺ T cells, regulatory T cells, and follicular helper T cells, in high-risk patients (Wilcoxon test). **J** Correlation analysis between CIKRRS scores and immune cell infiltration levels (Spearman test). **K**, **L** Box plots and radar plots of cancer-immunity cycle activity scores indicating enhanced antigen release and presentation, immune cell recruitment, infiltration, and T cell recognition in high-risk patients (Wilcoxon and Spearman tests). **M** Cytolytic activity scores comparing risk groups, showing significantly higher activity in high-risk patients (Wilcoxon test). **N**, **O** Predicted drug sensitivity (IC50) and correlations with CIKRRS for sunitinib, temsirolimus, and sorafenib (Wilcoxon and Spearman tests). *P < 0.05, **P < 0.01, ***P < 0.001

Analysis of somatic mutation profiles revealed that BAP1, SETD2, and MTOR mutations were more frequent in the high-risk group, with mutation rates of 18%, 18%, and 10%, respectively, compared with rates below 10% in the low-risk subset (Fig. 6A-B). Significant patterns of mutational co-occurrence were observed among high-risk patients, including VHL with PBRM1 and AHNAK2 with MUC16, as well as between SETD2 and PBRM1 in another subgroup (*P* < 0.05, Fig. 6C-D). Consistently, high-risk patients exhibited significantly elevated HRD scores across all metrics (*P* < 0.05, Fig. 6E), which were positively correlated with CIKRRS (*P* < 0.01, Fig. 6F). Moreover, patients with both high TMB and high CIKRRS demonstrated the poorest overall survival (*P* < 0.001, Fig. 6G).

In the tumor immune microenvironment, high-risk tumors showed significantly higher ImmuneScore and ESTIMATEScore, together with increased infiltration of CD8⁺ T cells, Tregs, and follicular helper T cells (Fig. 6H-J). Consistent with this immune-inflamed phenotype, CIKRRS was positively associated with multiple steps of the cancer-immunity cycle, particularly T cell recruitment (Fig. 6K-L), and with higher cytolytic activity (Fig. 6M). In silico drug sensitivity analyses further suggested lower estimated IC50 values for sunitinib and temsirolimus, but higher values for sorafenib, in the high-risk group (Fig. 6N-O).

### TNFSF14 links cytotoxic T- and NK cell immunity in ccRCC

**Fig. 7:**
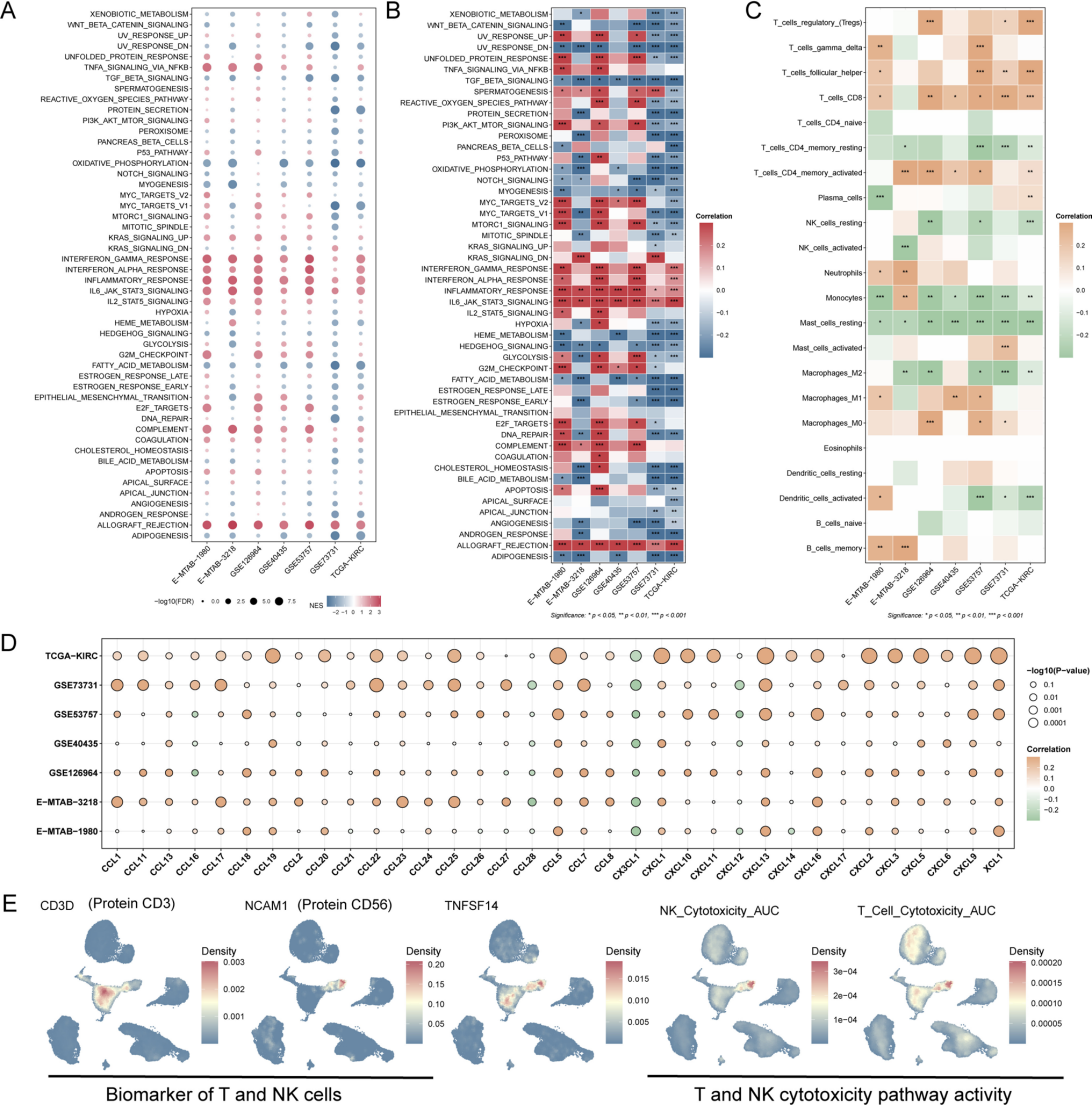
TNFSF14-associated immune features in ccRCC **A** Gene set enrichment analysis (GSEA) stratified by TNFSF14 expression across multiple ccRCC cohorts, showing significant enrichment of IFN-α response, IFN-γ response, inflammatory response, IL-6-JAK-STAT3 signaling, and allograft rejection pathways in tumors with high TNFSF14 expression. **B** Correlation analysis between TNFSF14 expression and Hallmark pathway activity scores, confirming positive associations with immune- and inflammation-related pathways and negative associations with metabolic programs across datasets. **C** Correlation heatmap showing associations between TNFSF14 expression and immune cell infiltration estimated by CIBERSORT, demonstrating positive correlations with CD8⁺ T cells, and activated CD4⁺ memory T cells. **D** Bubble plot illustrates correlations between TNFSF14 expression and chemokines involved in lymphocyte recruitment, including CCL5, CXCL9, CXCL10, CXCL11, and CXCL16, across independent ccRCC datasets, with dot size representing -log10(*P* value) and color indicating correlation strength. **E** Single-cell RNA-sequencing-based feature plots showing the distribution of CD3D (T cell marker), NCAM1 (NK cell marker), and TNFSF14 expression, together with NK cell and T cell cytotoxicity activity scores (AUCell), highlighting enrichment of TNFSF14 within cytotoxic T- and NK cell clusters and spatial overlap with regions of high cytotoxic activity. *P < 0.05, **P < 0.01, ***P < 0.001

Based on the RSF analysis of CIKRRS genes, TNFSF14 was identified as a key gene associated with survival in patients with ccRCC (**Fig. S3**). We further benchmarked TNFSF14 as a single-gene marker against the multigene CIKRRS score. In both TCGA-KIRC and E-MTAB-1980 cohorts, CIKRRS showed stronger prognostic discrimination than TNFSF14, with higher C-index values (0.792 vs 0.618 in TCGA-KIRC; 0.737 vs 0.666 in E-MTAB-1980) and consistently higher time-dependent AUCs (**Table S9**). These results support the added value of the multigene CIKRRS model for risk stratification, while TNFSF14 was prioritized for mechanistic follow-up. To further investigate the role of TNFSF14 in renal cancer immunotherapy, we performed integrative analyses across multiple datasets at both bulk and single-cell levels. At the bulk transcriptomic level, GSEA stratified by TNFSF14 expression (TNFSF14-high/low defined as the top/bottom 30% of TNFSF14 expression within each cohort) revealed that tumors with high TNFSF14 expression exhibited significant enrichment of IFN-α and IFN-γ response pathways, inflammatory response, IL-6-JAK-STAT3 signaling, and allograft rejection programs, collectively indicating a highly activated, immune-inflamed tumor microenvironment dominated by cytotoxic T- and NK cell-mediated immune responses (Fig. 7A). Consistently, correlation analyses between TNFSF14 expression and pathway activity further supported these findings (Fig. 7B).

Across datasets, TNFSF14 expression was positively associated with infiltration of cytotoxic and antigen-experienced T cell populations, as well as with key chemokines involved in T- and NK cell recruitment, including CCL5, CXCL9, CXCL10, CXCL11, and CXCL16 (Fig. 7C-D). At the single-cell level, TNFSF14 was enriched in T- and NK cell populations and spatially overlapped with regions of high cytotoxicity AUCell scores, further supporting its association with a CIK expansion-induced cytotoxic program (Fig. 7E).

### Phenotypic characterization of CIK cells

**Fig. 8:**
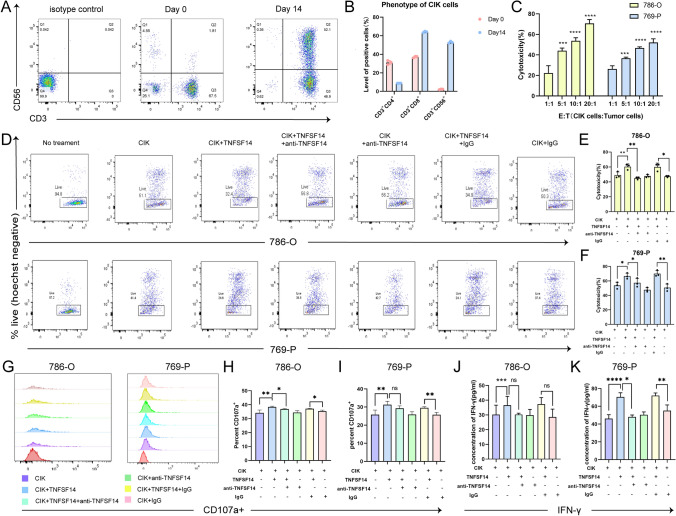
Phenotypic characterization of CIK cells and TNFSF14-mediated enhancement of cytotoxic function **A**, **B** Flow cytometry plots **A** and **B** show the immunophenotypes of CIK cells at different time points during in vitro culture. **C** Schematic diagram and quantitative analysis of CIK cells co-cultured with CFSE-labeled renal cancer cell lines (786-O and 769-P) at the indicated effector-to-target cell (E/T) ratio for 24 h. **D** Representative flow cytometry plots showing tumor cell viability when co-cultured with 786-O and 769-P cells under different experimental conditions. **E**, **F** Quantitative analysis of tumor cell viability when co-cultured with 786-O **E** and 769-P **F** cells under the indicated experimental conditions. **G** Representative flow cytometry plots showing CD107a expression in CD3⁺CD56⁺ CIK cell populations under different experimental conditions. **H**, **I** Quantitative analysis of CD107a expression in CD3⁺CD56⁺ CIK cell populations when co-cultured with 786-O **H** and 769-P **I** cells under the indicated experimental conditions. **J**, **K** The levels of IFN-γ in the supernatant collected after co-culturing CIK cells with 786-O **J** or 769-P **K** cells were detected by enzyme-linked immunosorbent assay (ELISA).*P < 0.05, **P < 0.01, ***P < 0.001, ****P < 0.0001

Phenotypic changes of CIK cells during in vitro expansion were assessed by flow cytometry. As shown in Fig. 8A-B, after 14 days of culture, the proportion of CD3⁺CD56⁺ NK-like T cells significantly increased from 2.23% ± 0.36% to 52.93% ± 1.04% (*P* < 0.0001), consistent with previously reported results [39], and these cells, along with CD8⁺ T cells, constituted the majority of the CIK cell population. **Figure S4A** illustrates the gating strategy.

### TNFSF14 enhances CIK-mediated cytotoxicity

The cytotoxic effect of CIK cells was assessed by co-culturing CFSE-stained renal cancer cells with CIK cells at varying effector-to-target ratios (1:1, 5:1, 10:1, and 20:1) for a duration of 24 h. Figure 8C illustrates that an E/T ratio of 10:1 led to cytotoxicity levels around 40–60%, which was then used for further experiments. To determine the optimal concentration of TNFSF14, increasing doses of TNFSF14 (0, 50, 100, and 200 ng/mL) were added to the co-culture system at an E/T ratio of 10:1. As shown in **Fig. S4B**, 100 ng/mL TNFSF14 produced the strongest enhancement of cytotoxicity and was selected for further analyses. As shown in Fig. 8D-F, baseline cytotoxicity of CIK cells against 786-O target cells was 49.00% ± 4.36%. Addition of TNFSF14 significantly increased cytotoxicity to 60.33% ± 3.79% (*P* < 0.01). In contrast, co-treatment with anti-TNFSF14 decreased cytotoxicity to 45.00% ± 1.73% (*P* < 0.01). The combination of TNFSF14 with isotype IgG maintained elevated cytotoxicity (60.00% ± 5.20%), whereas isotype IgG alone resulted in lower cytotoxic activity (47.33% ± 1.15%, *P* < 0.05). Comparable outcomes were seen in 769-P cells. Together, these data indicate that TNFSF14 increases CIK-mediated cytotoxicity against renal cancer cells, which can be attenuated by TNFSF14 neutralization.

#### TNFSF14 increases CIK cell degranulation and IFN-γ production

CIK cell degranulation and cytokine secretion were evaluated as functional correlates of CIK cytotoxic activity. Degranulation activity was defined as the percentage of CD107a⁺ cells within the CD3⁺CD56⁺ population. As shown in Fig. 8G-I, TNFSF14 significantly increased the proportion of CD107a⁺ CIK cells compared with untreated controls (786-O: 38.37% ± 0.55% vs 34.13% ± 2.06%; 769-P: 31.30% ± 1.90% vs 25.87% ± 2.46%). This increase was reduced by the addition of anti-TNFSF14. Compared with the isotype IgG control, co-treatment with TNFSF14 and IgG maintained higher degranulation levels. Consistently, following 24-h co-culture with tumor cells, TNFSF14 increased IFN-γ secretion by CIK cells (786-O: 36.62 ± 5.97 pg/mL vs 30.28 ± 6.36 pg/mL, *P* < 0.01; 769-P: 70.45 ± 4.77 pg/mL vs 46.15 ± 4.41 pg/mL, *P* < 0.01). This increase was reduced by anti-TNFSF14, while IFN-γ levels remained elevated in the presence of isotype IgG controls (Fig. 8J-K).

## Discussion

Given the aggressive behavior and poor prognosis of ccRCC, there is a continued need for improved immunotherapeutic strategies and robust risk stratification [40]. CIK cell-based therapy has shown promise in ccRCC [41], highlighting the importance of identifying molecular determinants linked to its efficacy and clinical relevance [10, 42]. In this study, we defined CIK-EIGs as transcriptomic changes induced during ex vivo CIK generation and used them to map related activation states across bulk and single-cell ccRCC datasets. Importantly, CIK-EIGs represent an ex vivo expansion-induced program rather than direct evidence of bona fide CIK cells in untreated tumors. Their enrichment in T/NK compartments, together with detectable activity in epithelial cells, suggests that the framework captures immune-inflamed and proliferative transcriptional states with partial overlap to canonical cytotoxic programs. Consistently, the bulk CIK-EIG score correlated with cytolytic activity in TCGA-KIRC, supporting its relevance to immune activation.

While CIK-EIGs provide a descriptive framework for immune activity, translating these transcriptional features into clinically actionable information requires outcome-oriented modeling. Accordingly, we constructed the CIKRRS, an optimized prognostic model derived from a reduced subset of CIK-associated genes selected for survival relevance. Previous studies have introduced a variety of immune cell-based prognostic models, including those derived from T cells, macrophages, and NK cells, which have demonstrated promising efficacy [43–45]. However, this work extends immune cell-based risk stratification frameworks to CIK cell-associated transcriptional characteristics. Previously, LASSO regression and Cox regression analyses have been frequently employed for prognosis-related modeling, demonstrating their robustness and efficacy [46–48]. In our study, we employed multiple survival-oriented machine learning approaches to construct CIKRRS and evaluated these models using the concordance index. In contrast to the descriptive CIK-EIG score, CIKRRS was designed to maximize prognostic discrimination and clinical applicability. Across independent cohorts, CIKRRS consistently stratified overall survival and remained informative within most clinicopathological subgroups. Tumors classified as high risk by CIKRRS exhibited pronounced immune infiltration, elevated cytolytic activity, and enrichment of inflammatory signaling pathways, including IFN-α/γ responses and IL6-JAK-STAT3 signaling. Importantly, however, the prognostic value of CIKRRS was not merely attributable to generic cytotoxicity, because riskScore remained independently associated with overall survival after adjustment for cytolytic activity. These findings indicate that CIKRRS captures a broader immune-inflamed yet clinically aggressive tumor state, in which cytotoxic activation coexists with biological programs associated with progression and poor outcome. Genomic analyses further showed that CIKRRS-high tumors were associated with increased homologous recombination deficiency and distinct somatic mutational patterns, supporting a complex interplay between genomic instability, inflammatory activation, and aggressive clinical behavior.

Among the genes contributing to CIKRRS, TNFSF14 emerged as a central determinant linking transcriptional signatures with functional immune activity. Previous bioinformatics analyses have reported that TNFSF14 is highly expressed in ccRCC and is associated with an unfavorable prognosis, suggesting a potential tumor-promoting role in ccRCC [49]. In our study, across bulk transcriptomic datasets, elevated TNFSF14 expression was strongly associated with cytotoxic immune infiltration, enhanced IFN-α and IFN-γ signaling, and increased activity across multiple steps of the cancer-immunity cycle. Single-cell analyses further localized TNFSF14 expression primarily to T and NK cell populations and demonstrated its concordance with regions of high cytotoxicity, indicating that TNFSF14 marks immune compartments functionally relevant to CIK-related activity. Importantly, functional assays supported a role for TNFSF14 in enhancing CIK cell effector function. Exogenous TNFSF14 significantly enhanced CIK-mediated tumor cell killing, promoted degranulation as measured by CD107a expression, and increased IFN-γ secretion, whereas neutralization of TNFSF14 attenuated these effects. These findings indicate that TNFSF14 acts as a potent amplifier of CIK cytotoxicity rather than a passive biomarker. This does not necessarily contradict the unfavorable prognosis associated with the broader immune-inflamed phenotype in vivo, because direct enhancement of CIK effector function in a controlled co-culture system may coexist with ineffective or restrained antitumor immunity in the complex suppressive tumor microenvironment.

Mechanistically, this functional enhancement is biologically plausible. Previous studies have characterized TNFSF14 as an important co-stimulatory ligand capable of promoting T cell activation and expansion and participating in the regulation of immune responses, including autoimmune processes [50, 51]. Upon engagement of its receptor HVEM, LIGHT has been shown to activate classical NF-κB signaling and engage the PI3K/Akt pathway, thereby supporting T cell survival and functional activation [52–54]. Consistent with these established signaling paradigms, our data suggests that TNFSF14 likely enhances CIK-mediated cytotoxic responses through similar intracellular pathways, reinforcing IFN-dominated inflammatory signaling and effective immune-tumor interactions, although receptor-specific mechanisms in CIK cells warrant further investigation. From a translational perspective, the dual role of TNFSF14 as both a prognostic component of CIKRRS and a functional enhancer of CIK activity highlights its therapeutic relevance. Modulating TNFSF14 signaling may represent a rational strategy to augment CIK-related immunotherapies in immune-inflamed ccRCC tumors. Collectively, these observations position TNFSF14 as a mechanistic bridge connecting transcriptional features, functional cytotoxicity, and clinical outcome. Nevertheless, the net clinical impact of TNFSF14 is likely to be context-dependent and shaped by the broader immune and stromal constraints of ccRCC tumors.

Compared with prior studies that primarily focused on phenotypic or functional characterization of CIK cells, our study establishes a transcriptomic framework linking an ex vivo CIK expansion-anchored program to tumor heterogeneity, prognostic modeling, and functional validation of TNFSF14-mediated enhancement of CIK cytotoxicity. Several limitations should nevertheless be considered. First, CIK-EIGs were derived from ex vivo expansion of healthy donor PBMCs and therefore represent an expansion-induced program; patient-derived CIK cells may exhibit additional disease-conditioned states. Second, because we did not directly compare ex vivo CIK cells with activated NK or T cell populations, CIK-EIGs should not be regarded as fully CIK-specific, but rather as a CIK expansion-anchored program with overlap with broader cytotoxic lymphocyte activation states. In addition, treatment annotations were not uniformly available across the public cohorts, limiting treatment-stratified analyses. Accordingly, although CIKRRS was associated with immune-inflamed features, cytolytic activity, and predicted drug sensitivity patterns, its treatment-specific predictive value for tyrosine kinase inhibitors or immune checkpoint blockade remains to be determined in well-annotated cohorts.

In summary, by linking CIK-EIG-anchored transcriptional characterization, CIKRRS-guided prognostic stratification, and functional assessment, this work identifies TNFSF14 as a mechanistically relevant modulator within the CIK-EIG framework and supports its potential relevance for optimizing CIK-based immunotherapeutic strategies in ccRCC.

## Supplementary Information

Below is the link to the electronic supplementary material.

Supplementary file1 (DOCX 692 KB)

Supplementary file2 (XLSX 220 KB)

## Data Availability

The RNA-sequencing data and corresponding clinical information, as well as mutation-related data, were downloaded from the Cancer Genome Atlas (TCGA) database (https://portal.gdc.cancer.gov/), the University of California Santa Cruz (UCSC) Genome Browser website (https://genome.ucsc.edu), and Gene Expression Omnibus (GEO) database. The dataset E-MTAB-1980 and E-MTAB-3218 cohort was acquired from the ArrayExpress database. The flowchart was created in the BioRender website (https://BioRender.com).
