# Supplementary material for: Cytokine-induced killer (CIK) Cells-associated transcriptome signature reveals the potential immunomodulatory role of TNFSF14 in clear cell renal cell carcinoma
Source: Cancer Immunol Immunother. 2026 May 20;75(6):167. doi: 10.1007/s00262-026-04422-y (PMC13212826; doi:10.1007/s00262-026-04422-y)
Supplement: Supplementary file 1 — Supplementary file1 (DOCX 692 KB) [file 262_2026_4422_MOESM1_ESM.docx]

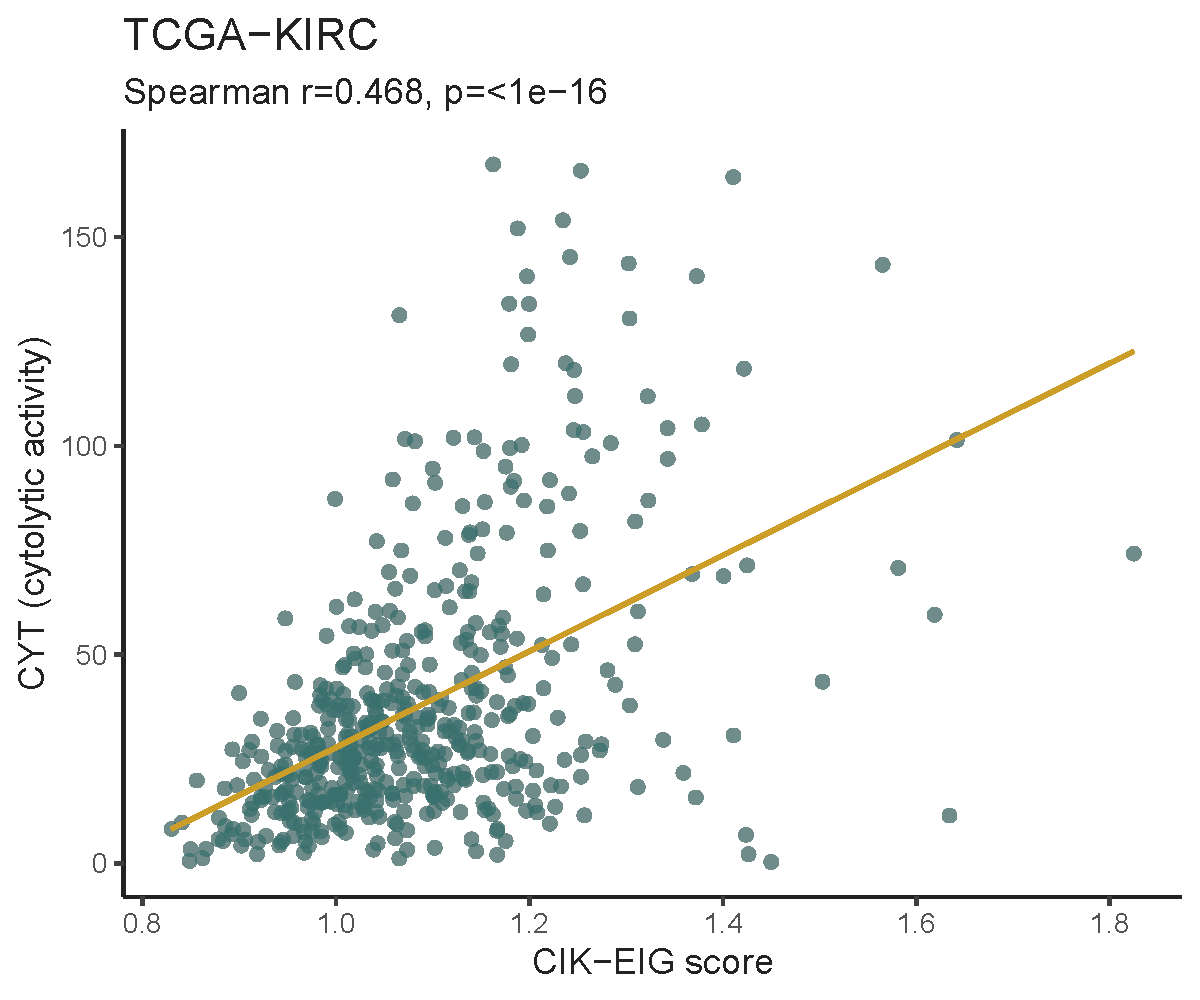


**Figure S1. Correlation between bulk CIK-EIG score and cytolytic activity in TCGA-KIRC.**

Scatter plot showing the association between the CIK-EIG score and cytolytic activity (CYT) in TCGA-KIRC samples. The CIK-EIG score was calculated by ssGSEA based on the CIK-EIG gene set. Spearman’s correlation coefficient and P value are shown in the panel.

**
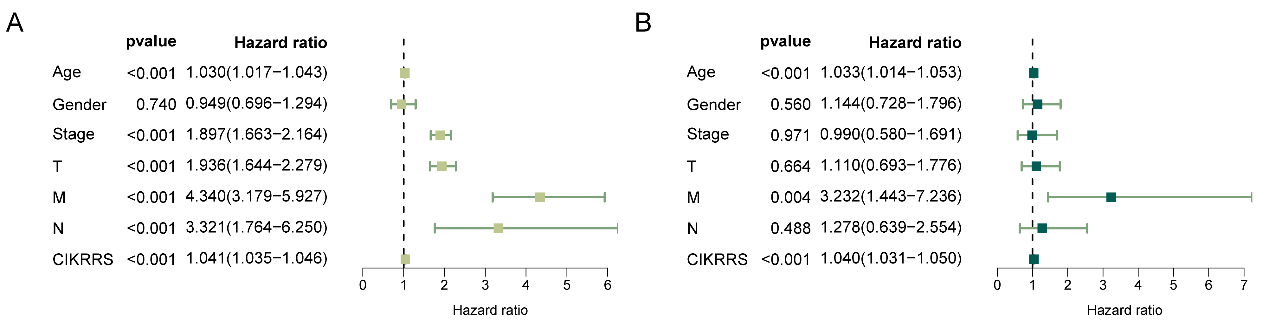
**

**Figure S2. Univariate (A) and multivariate (B) Cox regression analyses of CIKRRS and clinical parameters in TCGA-KIRC database.**

**
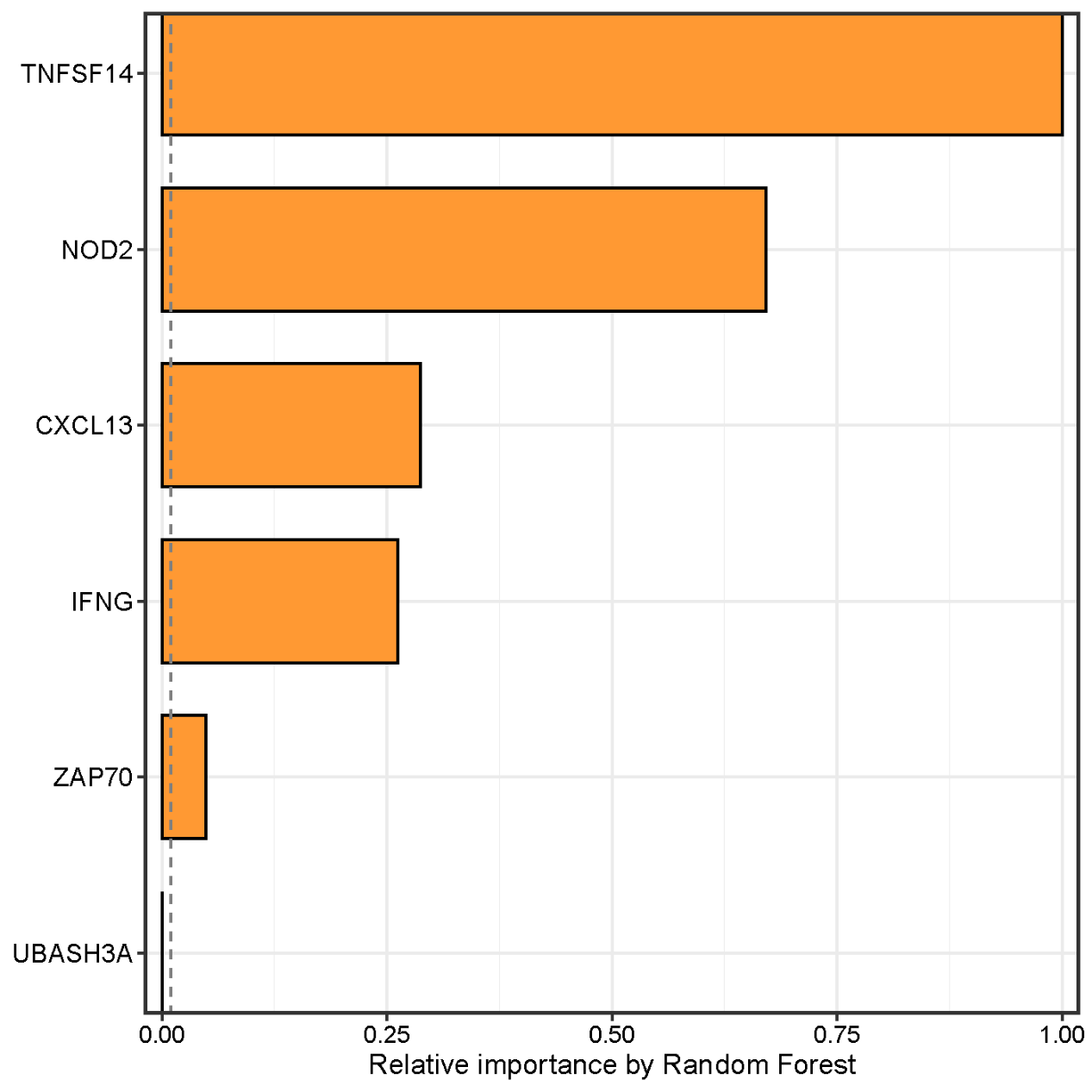
**

**Figure S3. Random Survival Forest (RSF) analysis of CIKRRS genes in TCGA-KIRC database, identified TNFSF14 as the key gene.**

**
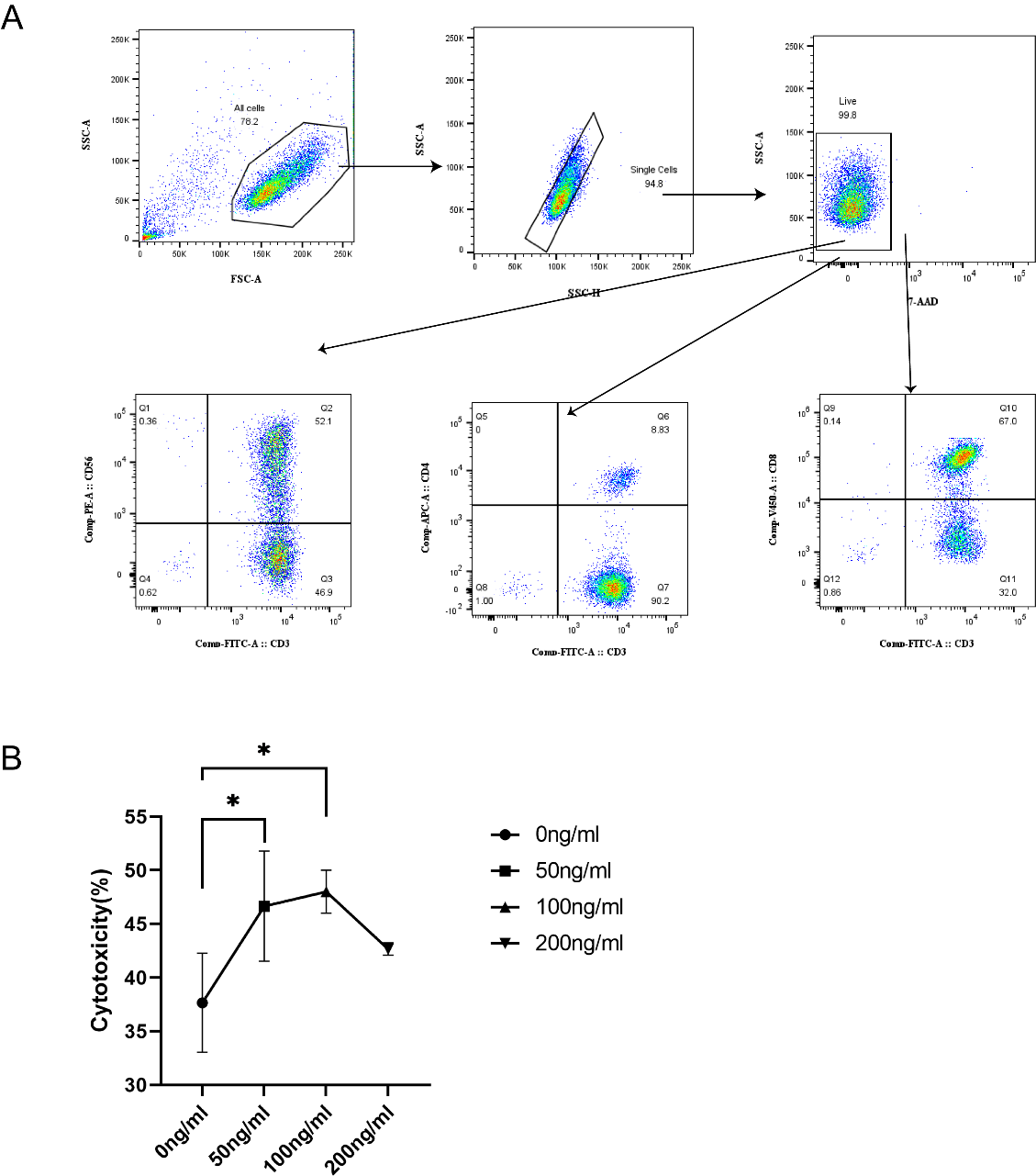
**

**Figure S4. (A) Gating strategy which was applied in phenotypic identification of CIK cells. (B) The cytotoxicity of CIK cells when cultured in different concentration of recombinant human TNFSF14 (LIGHT) protein.**
